# Genome sequence of the oleaginous yeast *Rhodotorula toruloides* strain CGMCC 2.1609

**DOI:** 10.1016/j.gdata.2017.05.009

**Published:** 2017-05-15

**Authors:** Christine Sambles, Sabine Middelhaufe, Darren Soanes, Dagmara Kolak, Thomas Lux, Karen Moore, Petra Matoušková, David Parker, Rob Lee, John Love, Stephen J. Aves

**Affiliations:** Biosciences, College of Life and Environmental Sciences, University of Exeter, Geoffrey Pope Building, Stocker Road, Exeter EX4 4QD, UK

**Keywords:** Aneuploid, Basidiomycota, Carotenoid, Oleaginous yeast, *Rhodosporidium toruloides*, Triacylglycerol

## Abstract

Most eukaryotic oleaginous species are yeasts and among them the basidiomycete red yeast, *Rhodotorula* (*Rhodosporidium*) *toruloides* (Pucciniomycotina) is known to produce high quantities of lipids when grown in nitrogen-limiting media, and has potential for biodiesel production. The genome of the CGMCC 2.1609 strain of this oleaginous red yeast was sequenced using a hybrid of Roche 454 and Illumina technology generating 13 × coverage. The *de novo* assembly was carried out using MIRA and scaffolded using MAQ and BAMBUS. The sequencing and assembly resulted in 365 scaffolds with total genome size of 33.4 Mb. The complete genome sequence of this strain was deposited in GenBank and the accession number is LKER00000000. The annotation is available on Figshare (doi:10.6084/m9.figshare.4754251).

**Table:** 

Specifications	
Organism/cell line/tissue	*Rhodotorula toruloides* strain CGMCC 2.1609
Sex	N/A
Sequencer or array type	Roche 454 & Illumina GAIIx
Data format	Raw data and analysed; *i.e.* assembly
Experimental factors	Genomic sequence of pure culture
Experimental features	Genomic sequence of pure culture
Consent	Not applicable. Data are available without restriction
Sample source location	Chinese General Microbiological Culture Collection (CGMCC)

## 1 Direct link to deposited data

https://www.ncbi.nlm.nih.gov/bioproject/PRJNA297267

https://figshare.com/articles/Gene_annotation_of_the_oleaginous_yeast_Rhodotorula_toruloides_strain_CGMCC_2_1609/4754251

## 2 Introduction

*Rhodotorula toruloides* (formerly *Rhodosporidium toruloides* [1]) is an oleaginous red yeast which can accumulate lipids to 75% of biomass [2], [3]. *R. toruloides* lipids are principally triacylglycerols comprising mostly C_16_ and C_18_ fatty acids [2], [4], [5] and biodiesel can be directly produced by methanolysis of dried cellular biomass [6]. *R. toruloides* can metabolise the five- and six-carbon sugars liberated by degradation of lignocellulosic biomass, and can achieve a high lipid yield [7], [8]. *R. toruloides* can also metabolise other economically relevant feedstocks including acetic acid [9], glycerol [10] and inulin [11], [12]. Here we report the genome sequence of *R. toruloides* strain CGMCC 2.1609 which has inulinase activity [11].

## 3 Experimental design, materials and methods

### 3.1 Culture conditions and DNA isolation

*R. toruloides* CGMCC 2.1609 was cultured at 28 °C in YMY medium (10 g l^− 1^ glucose, 5 g l^− 1^ soybean peptone, 3 g l^− 1^ yeast extract, 3 g l^− 1^ malt extract) for three days. Cells from 5 ml culture were resuspended in 1 ml of 50 mM citrate/phosphate pH 5.6, 40 mM EDTA, 1.2 M sorbitol, plus 2.5 mg Zymolyase, incubated at 37 °C for 60 min, washed in 0.55 ml of TE (10 mM Tris, 1 mM EDTA pH 8.0) containing 10 g l^− 1^ SDS and incubated at 65 °C for 60 min. 175 μl of 5 M potassium acetate was added and the sample kept on ice for 5 min, centrifuged (4 °C, 15 min) and DNA was precipitated from the supernatant with an equal volume of ice-cold isopropanol at − 20 °C for 60 min, washed twice with 70% ethanol, and incubated in 350 μl TE containing 50 mg l^− 1^ ribonuclease A at 65 °C for 10 min. After two phenol/chloroform extractions the DNA was precipitated with 70% ethanol on ice for 10 min, washed with 70% ethanol and resuspended in 30 μl TE.

### 3.2 Preparation of 2 kb and 4 kb mate pair libraries for genome sequencing

DNA quantification and quality control were performed by agarose gel electrophoresis and measurement of the absorbance at 260 nm, 280 nm and 230 nm confirming an OD 260/280 of between 1.8 and 2. The mate pair libraries were prepared according to the Illumina protocol (Preparing 2–5 kb Samples for Mate Pair Library Sequencing; Part # 1005363 Rev. B; February 2009) with a few modifications. DNA shearing was optimised using a Nebulizer and all DNA purification steps up to the circularisation of the fragments were carried out using a Qiagen Qiaex II gel extraction kit under the conditions described in the Illumina Mate Pair Library V2 kit (Part # 15008135, November 2009). Quality, quantity and size distribution of the purified DNA were checked using an Agilent Bioanalyzer DNA12000 chip for 2 and 4 kb fragments and a DNA 1000 chip for fragment size between 350 and 650 bp.

### 3.3 Genome sequencing

Genome sequencing was performed using a combination of single-read Roche 454 Life Sciences GSFLX Titanium (Liverpool Advanced Genomics Facility) and 2 kb and 4 kb mate pair Illumina GAIIx technology (Exeter University Sequencing Service). Two rounds of 454 sequencing provided a total of 2,248,095 reads (~ 843 Mb). *De novo* assembly of trimmed reads was performed using MIRA version 2.9.43 [13] followed by filtering of small and low coverage contigs (< 5 kb; coverage > 10 ×). Scaffolding of contigs was assisted by Illumina sequencing of 2 kb and 4 kb mate-pair libraries using Maq/Bambus [14]. CGMCC 2.1609 assembled as 365 scaffolds with an average coverage of 13 × and a total size of 33.4 Mb. Using in-house RNA-seq and proteomic data as additional evidence, 9820 gene models were predicted using MAKER and were functionally annotated with annot8r [15].

**Table:** 

General features of *Rhodotorula toruloides* CGMCC 2.1609 genome.	
Number of scaffolds	365
Number of contigs	868
Contig N50	65,782
Scaffold N50	190,017
Predicted coding sequences (CDS)	9820
GC content (%)	61.9
Size (bp)	33.4 Mb
≥ 99.0% identity with genome of *MAT-A1* haploid CBS 14	18.4 Mb
≥ 99.0% identity with genome of *MAT-A2* haploid CBS 349	13.9 Mb

The genome of *R. toruloides* strain CGMCC 2.1609 assembles as 33.4 Mb, which is larger than the ~ 20.3 Mb sequenced genomes of haploid *R. toruloides* strains [16], [17], [18], [19]. Of the assembled CGMCC 2.1609 genome, 18.4 Mb (55.1%) is highly similar (≥ 99% identity) to the genome of A1 mating-type haploid strain CBS 14 (ATCC 10788; IFO 0559), whereas 13.9 Mb (41.6%) is highly similar to the genome of A2 mating-type haploid CBS 349 (ATCC 10657; IFO 0880). These two haploid strains of *R. toruloides* are very divergent [16], sharing only 87% identity in genomic nucleotide sequences, despite the fact that they can mate as exemplified by the type strain of *R. toruloides* [20]. The CGMCC 2.1609 genome sequenced here contains only A1 mating-type information [21], [22], which is consistent with aneuploidy observed in progeny from such a mating [23].
